# Equal nano-characteristics, unequal harm: the chemical composition of noble metal nanoparticles as the main factor of cytotoxicity

**DOI:** 10.3389/ftox.2026.1812785

**Published:** 2026-05-18

**Authors:** Antónia Kurillová, Lucie Suchánková, Anastassiya Pedan, Šárka Hradilová, Lukáš Malina, Kateřina Bartoň Tománková, Barbora Hošíková, Jana Zapletalová, Hana Kolářová, Libor Kvítek, Aleš Panáček

**Affiliations:** 1 Department of Physical Chemistry, Faculty of Science, Palacký University Olomouc, Olomouc, Czechia; 2 Regional Centre of Advanced Technologies and Materials, Czech Advanced Technology and Research Institute (CATRIN), Palacký University, Olomouc, Czechia; 3 Department of Medical Biophysics, Faculty of Medicine and Dentistry, Palacký University Olomouc, Olomouc, Czechia; 4 Institute of Molecular and Translational Medicine, Palacký University Olomouc, Olomouc, Czechia

**Keywords:** apoptosis, cytotoxicity, DNA damage, metal nanoparticles, necrosis, ROS

## Abstract

**Background:**

Metal nanoparticles are increasingly explored in biomedical and technological applications, yet their cytotoxicity remains difficult to interpret due to the strong influence of multiple physicochemical parameters. Differences in synthesis protocols, particle size, morphology, and surface properties across studies often hinder direct comparison of toxicological outcomes and limit the ability to attribute observed effects to the chemical composition of the nanoparticle core.

**Methods:**

In this study, silver (Ag), copper (Cu), and gold (Au) NPs were synthesized under identical conditions to control for size, shape, and surface charge, thereby isolating the effect of chemical composition. The NPs (8–9 nm, spherical, zeta potential ∼ –21 mV) were evaluated in NIH 3T3 fibroblasts using MTT viability assays, reactive oxygen species (ROS) detection, mitochondrial membrane potential analysis, apoptosis quantification, and comet assays.

**Results:**

Ag NPs showed the highest toxicity (IC_50_ = 11.9 mg·L^-1^), followed by Cu NPs (51.6 mg·L^-1^), while Au NPs were the least toxic (228.2 mg·L^-1^). Mechanistic data revealed that Ag NPs induced severe oxidative stress and mitochondrial dysfunction, leading to apoptosis at sublethal concentrations and necrosis at higher doses. Cu NPs triggered strong ROS generation and apoptotic signaling. Au NPs showed minimal toxicity, with weak apoptotic effects only at the highest concentrations. None of the tested NPs caused significant DNA damage. Notably, cytotoxicity correlated with increased necrosis at higher doses and apoptosis at lower concentrations, indicating dose- and composition-dependent cell death mechanisms. While reactive oxygen species contributed to toxicity, the small size (∼8–10 nm), spherical morphology, and absence of surface modification enhanced cellular uptake and cytotoxic potential.

**Conclusion:**

Our results provide clear evidence that, for controlled nanoparticle properties, the toxicity hierarchy primarily reflects intrinsic chemical identity. These findings underscore the importance of separating nanoparticle composition from other particle-related artifacts, thereby supporting the rational design of safer biomedical nanomaterials through the controlled adjustment of size, surface chemistry, and metal composition.

## Introduction

Nanotechnology is one of the fastest-growing and most promising technologies, opening new possibilities for industrial, technological, or medical applications due to the remarkable properties of nanomaterials, such as electronic, optical, chemical, or biological properties (Jomova et al., 2025). With the growing interest in nanomaterials and their applications, nanoparticles of various chemical compositions, sizes, and morphology and nanomaterial-based commercial products for the automotive, construction, electronic, cosmetic, or medical industries have been produced during the last decade. On the other hand, this exciting technological progress may also bring risks as increasing nanoparticle production leads to higher exposure of living organisms and the environment to engineered nanomaterials (ENM). Such excessive exposure may pose health and environmental threats due to the still-unexplored biological behavior of nanoparticles (Giese et al., 2018; Kumah et al., 2023).

Noble metal nanoparticles represent one of the most important and frequently used groups of engineered nanoparticles. It is due to their unique physical (optical, electronic, and magnetic properties), catalytic, and biological properties (antimicrobial, anti-inflammatory and anticancer activity), which significantly differ from those of bulk material (Medici et al., 2021; Niżnik et al., 2024; Zafar et al., 2025). Biological properties, including toxicity of noble metal nanoparticles, are generally determined by their size, size distribution, shape, surface area, surface chemistry, surface charge, stability in the environment, and ability to release metal ions (Rajan et al., 2022). Therefore, the biological behavior of nanoparticles and their possible adverse effects cannot be derived in all cases, neither from a single particle characteristics (Sati et al., 2026) nor from the bulk form of material. Nanoparticles show unique properties and interactions with biological systems just due to their nanodimensions (Niżnik et al., 2024).

Silver NPs represent one of the most extensively studied groups of noble metal nanomaterials in terms of their biological properties and applications, largely due to their strong antimicrobial activity. Interestingly, prokaryotic cells (bacteria) are more sensitive to silver NPs than eukaryotic cells. 19% of all applications of silver nanoparticles are related to silver´s antibacterial properties (Bondarenko et al., 2013). Minimal inhibition concentration values for Ag NPs to bacteria are mostly present in the range 0.1–20 mg·L^-1^ (Składanowski et al., 2016; Kakian et al., 2024), while cytotoxic concentrations to eukaryotic cells varied from 10 to 100 mg·L^-1^ (Składanowski et al., 2016; Muhamad et al., 2022; Barbasz et al., 2015), depending on the nanoparticle size, morphology, and surface modification. The toxic properties of silver nanoparticles have been intensively studied, although it is challenging to generalize the results too broadly. However, most of the following generally apply: (i) smaller nanoparticles are more toxic than larger ones (Egbuna et al., 2021; Bélteky et al., 2021), (ii) small particles with a relatively larger surface area release more Ag^+^ ions, contributing to toxicity directly or through a Trojan horse mechanism (McNeilly et al., 2025), (iii) toxicity to mammalian cells is considerably lower in comparison with antibacterial effective concentrations (Medici et al., 2021; Hosny et al., 2025) due to the fact that eukaryotic cells have antioxidant cellular mechanism that protects them (Hosny et al., 2025). Some studies claim that the Ag NPs cytotoxicity is independent of the ions release (Wang et al., 2022), while others argue that it is the interaction of both nanoparticles and ions (Bidan and Al-Ali, 2024; Ameh and Sayes, 2019).

Gold is generally considered inert, low-toxic, and biocompatible, and because its nanoparticles share this low cytotoxicity toward eukaryotic cells, they can be used in a wide range of biological applications without risking cell damage (Aguilar-Garay et al., 2024; Lee et al., 2014). Especially, surface-modified and functionalized gold NPs are destined for use in imaging and detection techniques as well as for the transport of drugs and other bioactive molecules inside the tissue or cells (Wang et al., 2013). Gold NPs have the ability to bind to DNA (Kasyanenko et al., 2019), block transcription (Mikhailova, 2021), and are used in gene therapy or cancer treatment. Gold nanoparticles exhibit a size- and shape-dependent cytotoxicity profile that is more pronounced than that of many other metal nanoparticles. Their exceptionally adaptable morphology and surface chemistry are particularly noteworthy, making their biological effects highly sensitive to subtle changes in particle geometry, functionalization, concentration, and exposure duration (Woźniak et al., 2017).

Copper compounds and copper NPs are not as biocompatible as gold nanoparticles, but they are still less toxic than silver nanoparticles. Copper nanoparticles are easily oxidized, accompanied by the release of copper ions, causing cytotoxicity. Kahru et al. (2008) suggest that in the case of metal-based nanoparticles and metal oxides in general, the release of metal ions may be the main factor influencing their toxicity. Among the most common mechanisms of copper nanoparticle toxicity are the Trojan horse effect, which releases copper ions inside the cell, and the induction of oxidative stress (Ma et al., 2022; Moschini et al., 2023). Chen et al. summarized similar results in animal models (Chen et al., 2013), and Minander’s group found that copper ions released from copper nanoparticles can damage DNA (Midander et al., 2009).

Overall, the cytotoxicity of these noble metals can be summarized by the fact that, for silver and copper nanoparticles, the generation of reactive oxygen species is the primary mechanism contributing to their toxicity toward cells. The mechanism of ROS generation is the ability of the nanoparticles to donate an electron to molecular oxygen, thereby producing a reactive superoxide radical. This, in turn, triggers radical reactions affecting cell survival and death, signaling, or differentiation (Flores-López et al., 2019). Silver nanoparticles can affect the mitochondrial respiratory chain by ROS formation, affecting ATP production and damaging DNA. Ag^+^ released from nanoparticles is able to bind to thiol groups in proteins and thereby alter their function or structure (Zhang et al., 2022). Copper nanoparticles can induce various biochemical processes upon contact with cells, including the formation of reactive oxygen species, photooxidation, lipid oxidation, and the conjugation of nanoparticles with proteins and other molecules. These covalent modifications of molecules induced by interaction with copper nanoparticles lead to oxidative stress (Ameh and Sayes, 2019; Riaz et al., 2025). In the case of gold nanoparticles, the resulting cytotoxicity, uptake, and associated *in vivo* distribution depends primarily on the particle size and also on the cell type in which the gold nanoparticles are present (Xia et al., 2019). Smaller nanoparticles induce a higher cytotoxic effect due to their better biodistribution and presence inside the cell nucleus, which leads to DNA damage (Sani et al., 2021).

Recently, hundreds of scientific reports on the cytotoxicity of metal nanoparticles have been published. However, it is difficult to make a general conclusion on the cytotoxic properties of noble metal nanoparticles because different cytotoxic effects have been reported based on various testing methods on different cell lines. These effects depend on differently synthesized metal nanoparticles having different particle characteristics, such as size, shape, zeta potential, and surface modification, that strongly affect biological interactions with cells. Here, we present a thorough evaluation of the cytotoxic effects of gold, copper and silver nanoparticles synthesized in the same way, with similar sizes, shapes, zeta potentials, and surface modifications, on a single cell line using a single cytotoxic assay. The innovative aspect of this study lies in the systematic isolation of the influence of the chemical identity of these noble metal nanoparticles on cytotoxicity, independent of other variables such as size, morphology, surface treatment, dispersion medium, cell line, or testing method. This approach allows for clear evaluation, comparison, and interpretation of their cytotoxic effects based solely on the metal’s chemical nature. Several cytotoxic procedures such as viability test (MTT), measurement of reactive oxygen species production (ROS), mitochondrial membrane potential assay (MMP) and comet assay for DNA analysis. Types of cell death were applied to obtain a wide and comprehensive view on biological interactions of noble metal NPs with living cells and discuss their biological effects to the cell-based solely on the chemical identity of noble metal NPs.

## Materials and methods

### Chemicals and biological material

Silver nitrate (99.9%, Sigma–Aldrich), gold (III) chloride trihydrate (99.9%, Sigma–Aldrich), copper (II) nitrate trihydrate (p.a. Penta), and sodium borohydride (p.a. Sigma–Aldrich) were used for the preparation of nanoparticles without any further purification. Prepared metal NPs were stabilized by polyacrylic acid of low molecular weight (Mw 1,200, 45% (w/w), Sigma–Aldrich). All the used solutions were prepared with deionized water (conductivity 0.05 μS·cm^–1^) from instrument Aqual 29 (Merci).

Mouse fibroblast cells NIH 3T3 cell line, Dulbecco’s Modified Eagle Medium (DMEM), phosphate buffered saline (PBS, pH 7.4, own preparation), 5–(and–6)–chloromethyl–2′,7′–dichlorodihydrofluorescein diacetate (CM–H_2_DCFDA, Invitrogen Co., United States), thiazolyl blue tetrazolium bromide (MTT, Sigma–Aldrich), 5,5′,6,6′–Tetrachloro–1,1′,3,3′–tetraethyl–imidacarbocyanine iodide (C_25_H_27_Cl_4_IN_4_, JC–1, Sigma–Aldrich), dimethyl sulfoxide (DMSO, Sigma–Aldrich), HMP agarose (Serva, Biotech, Czech Republic), LMP agarose (Qbiogene, Genetica, Czech Republic), trypsin–EDTA (Sigma–Aldrich), ethanol (Sigma–Aldrich), fetal bovine serum (FBS, Sigma–Aldrich), NaCl (Tamda, Czech Republic), EDTA (ethylenediaminetetraacetic acid, Lachema, Czech Republic), tris (tris(hydroxymethyl) aminomethane, Sigma–Aldrich), triton X–100 (Serva), NaOH (Sigma–Aldrich), SYBR green (Invitrogen Co., United States), anti–phospho–histone H3 (Millipore), alexa fluor 488 goat anti–rabbit IgG (Molecular probes), propidium iodide (Sigma–Aldrich), ribonuclease A (Sigma–Aldrich). Measurements were carried out on the multi–detection microplate reader Synergy HT (BioTek, United States), transmission microscope Olympus IX81 with DSU unit (Olympus, Japan) and electrophoretic tank (Bio–RAD, Czech Republic). We used Phototox Version 2.0 (ZEBET, Germany) and Comet Score (Tritek Corp) as software. Program Phototox 2.0 determined the IC_50_ value, which was also used in other assays.

### Synthesis and characterization of noble metal nanoparticles

Nanoparticles of noble metals used in this work were synthesized by reduction of appropriate metal salt precursor via NaBH_4_. All the prepared metal NPs were stabilized by sodium salt of polyacrylic acid. Silver, gold, and copper nanoparticles were synthesized by reduction of silver nitrate, gold (III) chloride trihydrate, and copper (II) nitrate trihydrate. The final concentrations of the reaction components were as follows: 0.93 · 10^−2^ mol L^–1^ AgNO_3_, 4.06 · 10^−3^ mol·L^–1^ gold (III) chloride trihydrate, and 1.58 · 10^−2^ mol·L^–1^copper (II) nitrate trihydrate. The water solution of the sodium salt of polyacrylic acid was added prior to the addition of the reducing agent at a final concentration of 0.1% w/w. Finally, a solution of NaBH_4_ was rapidly added to the reaction mixture. The same concentration of the reducing agent equal to 10^−2^ mol·L^–1^ was used for all the synthesized noble metal NPs. All the reaction components were added to the reaction mixture under vigorous stirring, and noble metal NPs were synthesized at laboratory temperature. The final concentrations of noble metals in the prepared nanoparticle dispersions were 1,000 mg·L^-1^ for Ag NPs and Cu NPs and 800 mg·L^-1^ for Au NPs. The pH was set identically at value 8 for all the dispersions by adding an appropriate amount of 0.01 mol·L^–1^ NaOH solution.

The average sizes and size distributions of the prepared noble metal NPs were determined by dynamic light scattering (DLS) method and zeta potentials using the electrophoretic mobility technique using the Zetasizer Nano ZS (Malvern) instrument. The nanodimension of the synthesized silver NPs was confirmed by transmission electron microscopy using the JEM–2010 (Jeol) and by UV–vis absorption spectroscopy with the Specord S 600 spectrophotometer (Analytik Jena AG). Prior to the absorption spectra measurement, dispersions were diluted to the concentrations of 10 mg·L^-1^ Ag NPs, 40 mg·L^-1^ Au NPs, and 20 mg·L^-1^ Cu NPs.

### Cytotoxicity evaluation

To evaluate the cytotoxic effect of Ag, Au, and Cu nanoparticles, including cytotoxic mechanism on NIH 3T3 cells, procedures such as MTT cell viability assay, measurement of reactive oxygen species production, mitochondrial membrane potential assay, comet assay, and cell cycle determination were used. Dispersions of metal nanoparticles were diluted to desired concentrations in DMEM medium containing cell culture. Cells were incubated for 24 h at 37 °C and 5% CO_2_. After incubation, cytotoxic parameters were evaluated and compared with negative control. Also, chemical components NaBH_4_, sodium salt of polyacrylic acid used in the chemical synthesis of metal NPs were evaluated for their cytotoxic effect. NaBH_4_ was used after its 15 min hydrolysis in water solution.

### MTT cell viability assay

The cytotoxic effect and IC_50_ of NPs on NIH 3T3 cells were determined using the MTT assay. After 24 h of cell cultivation with metal NPs, DMEM was replaced by PBS prior to starting the MTT measurements, 50 μL of 20 mM MTT (dissolved in PBS) was added, and the cells were incubated for 3 h at 37 °C and 5% CO_2_. The MTT solution was carefully removed, and 100 μL DMSO was added in order to solubilize the violet formazan crystals. The absorbance of the resulting solution was measured in 96–well microplate reader Synergy HT at 570 nm and 690 nm. The cell viability of the samples was determined as a percentage of control cell viability (100 × average of test group/average of control group). Data were calculated using the Phototox v.2 software for the determination of IC_50_.

Tested concentrations: 100–1 mg·L^-1^ for Ag NPs, 800–12 mg·L^-1^ for Au NPs, and 1.000–10 mg·L^-1^ for Cu NPs.

### Measurement of reactive oxygen species production

Immediately after the addition of NPs to the 96 well plates with PBS, the ROS kinetic production was determined using 2 µL 500 mM CM–H2DCFDA fluorescence probes and microplate reader Synergy HT. Excitation wavelengths of 485 nm and emission wavelengths of 548 nm were used. Kinetic ROS production was detected immediately after the addition of nanoparticles to the cell line NIH 3T3 in 1-min intervals during 4 h of measurement. The time of CM–H_2_DCFDA probe incubation was 20 min before adding of NPs. Effects of metal nanoparticles on kinetic production of reactive oxygen forms (hydrogen peroxide H_2_O_2_, hydroxyl radical ·OH^−^, hypochlorous acid HOCl, and peroxyl radical ROO·) were expressed as regression coefficients of relative fluorescence unit (RFU) from the linear part of the curve.

Tested concentrations: 100–1 mg·L^-1^ for Ag NPs, 800–12 mg·L^-1^ for Au NPs, and 1.000–10 mg·L^-1^ for Cu NPs. The linear regression of the ROS rate expressed the amount of ROS created at each minute. Data represent the mean and standard error from three independent measurements.

### Mitochondrial membrane potential assay ΔΨm

Mitochondrial membrane potential (MMP) change was monitored by the fluorescent cationic voltage-dependent dye JC–1. NIH 3T3 cells were loaded in PBS with JC–1 (5 μg·mL^-1^, dissolved in DMSO) for 20 min at 37 °C, 5% CO_2_, and then washed with PBS twice. Results were expressed as the ratio of the fluorescence retained within the cells in green and red spectra. Change of mitochondrial membrane potential (ΔΨm changes) was determined 6 h after application of nanoparticles. It was determined the ratio of the median fluorescence of the green component of the monomer, which is located in damaged cells, to the median fluorescence of the red component unit, which is located mainly in the undamaged or control cells. Loss of mitochondrial membrane potential is a key feature of the early stages of apoptosis. Although this condition may ultimately result in necrosis, it also facilitates the identification of whether a given compound induces mitochondrial damage or triggers cell death through an alternative pathway. The higher the ratio of the monomer fluorescence of the green/red unit, the greater the damage to the mitochondria/cells and the greater the probability of the early stage of apoptosis.

Tested concentrations: 100–1 mg·L^-1^ for Ag NPs, 800–12 mg·L^-1^ for Au NPs, and 1.000–10 mg·L^-1^ for Cu NPs. The ratio of the median of green monomer and red aggregate expressed the amount created in dependence on the NPs concentration. The higher the values, the higher the probability of the early stage of apoptosis. The data represent the mean and standard errors from three independent measurements.

### Comet assay

Cells were incubated with appropriate concentrations (equal IC_50_ and 2× IC_50_) of metal nanoparticles for 6 h. The comet method from our previous study (Tomankova et al., 2011) was used. Briefly, microscope slides were first pre-coated with 1% HMP agarose. The cells were trypsinized, rinsed by DMEM with 10% FBS, and centrifuged (3 min, 1,500 rpm). A quantity of 85 μL of 1% LMP agarose was added to the 25 μL of cells pellet suspension, and 85 μL of this was added to the microscope slide with agarose gel. The microscope slides were immersed in a lysis buffer for 1h, then placed in an electrophoretic tank and dipped into a cool electrophoresis solution for 40 min. Electrophoresis was run at 20 V and 350 mA for 20 min. After neutralization in the buffer (0.4 M Tris, pH = 7.5), the samples were then stained by SYBR Green and imaged using the microscope Olympus IX81 with DSU unit. The images were then scored by SW Comet Score. Undamaged DNA moves slowly in the electric field and forms the so-called head of the comet, while DNA breaks are agile and create the so-called comet’s tail. The greater the amount of DNA in the tail, the larger the number of DNA breaks. After staining DNA and calculating fluorescence, the extent of DNA damage was determined. The length and fluorescence intensity of the comet tail relative to the comet head reflects the number of DNA breaks.

### Apoptotic/necrotic assay

The measurement was carried out using the Annexin V–Cy3 kit. Cells were incubated at 37 °C and 5% CO_2_ for 24 h in fresh DMEM together with NPs at an ½ IC_50_, IC_50_ and 2× IC_50_ concentration. Before starting the measurements, DMEM was replaced by a 50 µL of solution consisting of distilled water, binding buffer, annexin, propidium iodide and incubated at 37 °C and 5% CO_2_ for 10 min. Annexin binds to phospholipids such as phosphatidylserine, which under normal conditions is localized to the inner part of the lipid bilayer. During apoptosis, however, the bilayer undergoes remodeling, resulting in the translocation of phosphatidylserine to the outer part, where it is recognized by the annexin probe and visualized as green fluorescence. Propidium iodide is capable of binding to cellular DNA, but this occurs only when the integrity of the plasma membrane is compromised—a characteristic feature of necrosis. Necrotic cells are therefore detected as red fluorescence. The imaged apoptotic and necrotic cells were subsequently counted manually and converted into percentages. The fluorescence signal was obtained using a transmission microscope, Olympus IX81, with a DSU unit.

### Statistical analysis

The data are presented as the means of three independent biological replicates (n = 3), each measured in triplicate. Significant differences among groups were determined by paired or unpaired two-sample t-tests. The values for each particle type were compared to the corresponding controls, which were run on the same days and in the same manner as the tested particles. The level of statistical significance was p < 0.05.

## Results

### Synthesis and characterization of noble metal nanoparticles

**FIGURE 1:**
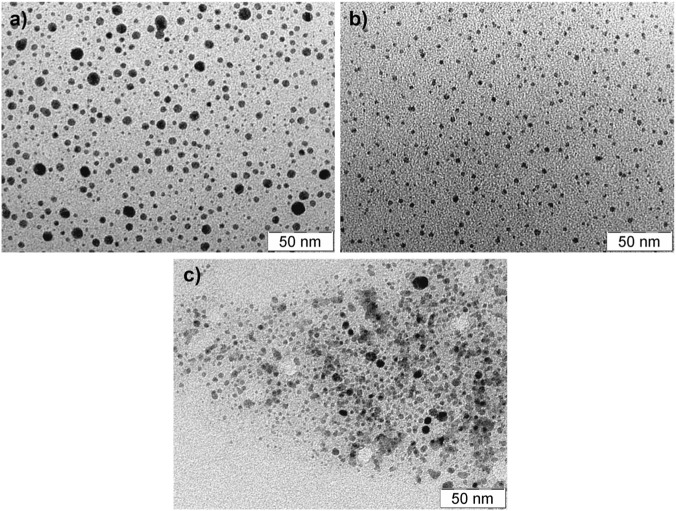
TEM images of **(a)** silver, **(b)** gold and **(c)** copper nanoparticles. All nanoparticles are spherical with an average size of 8–9 nm, showing a uniform size distribution. The scale bar represents 50 nm. These images confirm the successful synthesis of small, monodisperse noble metal nanoparticles, which were used for subsequent cytotoxicity studies.

**FIGURE 2:**
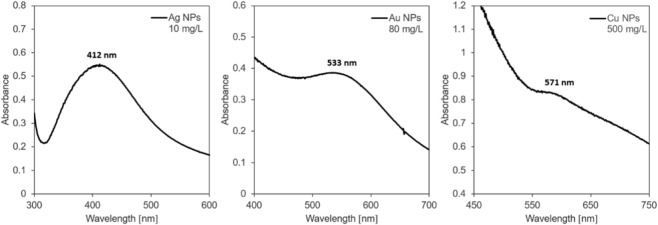
UV–Vis absorption spectra with surface plasmon bands characteristic of Ag, Au, and Cu nanoparticles.

Silver, gold, and copper nanoparticles were synthesized under identical reaction conditions using the same reduction method and the same reducing and stabilizing agent. Using this method, Cu, Ag, and Au NPs with similar characteristics were synthesized. The average size of the prepared noble metal NPs determined by DLS was 8, 8, and 9 nm, for silver, gold, and copper NPs, respectively. The size distributions of silver, gold, and copper NPs obtained from DLS measurements were also similar and ranged from 5 to 12 nm, 5–10 nm, and 6–10 nm for silver, gold, and copper NPs, respectively. The measured zeta potential values were also very similar, reaching values of −21 mV, −20 mV, and −23 mV for Ag NPs, Cu NPs, and Au NPs, respectively. All the synthesized metal NPs were of spherical shape. The size and spherical shape of the synthesized metal NPs were confirmed by transmission electron microscopy measurements (Figure 1). The presence of metal nanoparticles in the dispersion was confirmed by UV–Vis absorption spectrophotometry (Figure 2). A typical absorption band called surface plasmon was recorded for all noble metal NPs at the wavelengths of 412 nm, 533 nm, and 571 nm for silver, gold, and copper NPs.

All the synthesized aqueous dispersions of noble metal NPs kept in closed flasks, preventing access to atmospheric oxygen, were stable and showed excellent aggregation and sedimentation stability for 1 week. The dispersion of copper NPs remained stable and resistant to dissolution for 5 days after synthesis, due to the presence of an excess reducing substance that maintained a reductive environment in the water dispersion, and to the adsorption layer of polyacrylic acid, which prevented oxygen from accessing the surface of the copper NPs.

#### MTT cell viability assay

**FIGURE 3:**
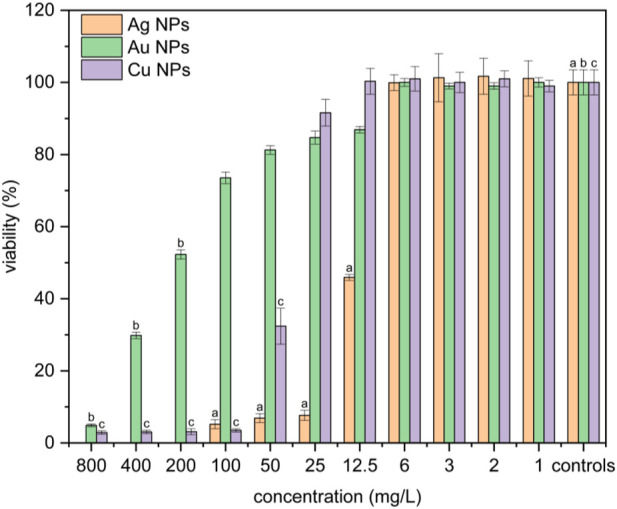
Viability of cells exposed to different Ag, Au, and Cu nanoparticle concentrations. Positive significance (a, b, c) was determined using ANOVA and the Dunnett *post hoc* test, p < 0.05 compared with the corresponding controls.

Cell viability was determined by the MTT assay using the NIH 3T3 cell line exposed to synthesized metal nanoparticles for 24 h. Silver nanoparticles exhibited the highest cytotoxicity compared to gold and copper nanoparticles (Figure 3). Cell viability significantly decreased for the concentrations of silver NPs ranging from 12 mg·L^-1^ to 100 mg·L^-1^. The cytotoxicity index IC_50_ for silver NPs was determined at a concentration of 11.9 mg·L^-1^ of Ag. Gold nanoparticles reduced the cells viability slightly, down to only 80% compared to control cells in the concentration range from 12.5 to 50 mg·L^-1^ of Au. The cytotoxic effect of gold NPs to NIH 3T3 was observed at the concentration range from 200 mg·L^-1^ to 800 mg·L^-1^. The IC_50_ index for gold nanoparticles was determined at a concentration of 228.23 mg·L^-1^. Copper nanoparticles showed a moderate cytotoxic effect compared to silver and gold NPs. The viability of cells exposed to Cu NPs significantly decreased at concentrations ranging from 50 mg·L^-1^ to 800 mg·L^-1^. The cytotoxicity index IC_50_ of Cu NPs was determined at a concentration of 51.6 mg·L^-1^.

**FIGURE 4:**
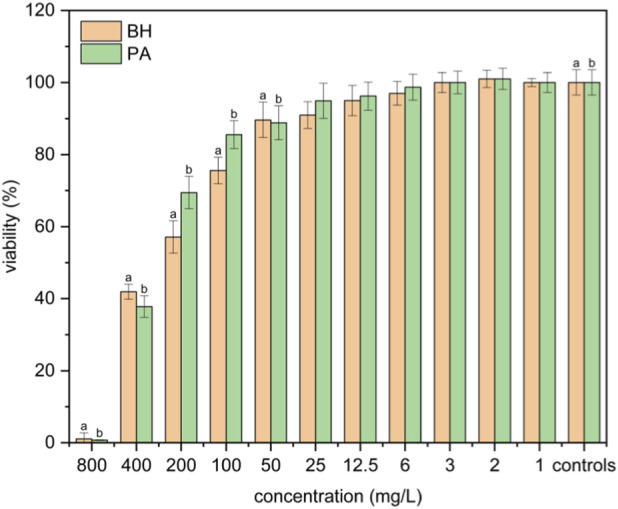
Viability of cells exposed to different concentrations of NaBH_4_ (BH) and polyacrylic acid (PA). Positive significance (a, b) was determined using ANOVA and the Dunnett *post hoc* test, p < 0.05 compared with the corresponding controls.

It must be noted that PA and BH showed cytotoxic effects for such high toxic concentrations of gold NPs (200 mg·L^-1^ and above). The viability of cells exposed to BH and PA decreased in a concentration range from 200 mg·L^-1^ to 800 mg·L^-1^, similarly to the case of gold NPs in the appropriate concentration interval of 400–800 mg·L^-1^. Neither PA nor BH showed toxic effects at concentrations ranging from 12 mg·L^-1^ to 100 mg·L^-1^, whereas silver NPs were toxic (Figure 4).

Determined IC_50_ for tested silver, gold, and copper NPs, including sodium borohydride and polyacrylic acid, were as follows: 11.9 mg·L^-1^ for Ag NPs, 228.2 mg·L^-1^ for Au NPs, 51.6 mg·L^-1^ for Cu NPs, 297.5 mg·L^-1^ for NaBH_4_, and 1.3 g·L^-1^ for polyacrylic acid.

### Measurement of reactive oxygen species production

**FIGURE 5:**
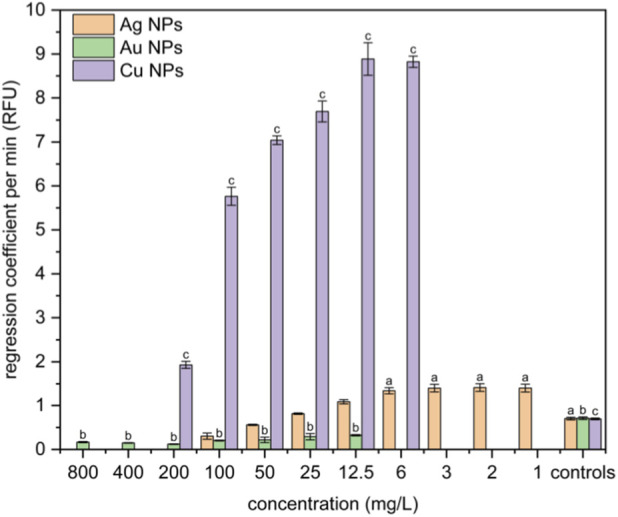
Kinetic production of ROS in NIH 3T3 cell line induced by Ag, Au, and Cu nanoparticles in concentrations from 1 to 800 mg·L^-1^. Positive significance (a, b, c) was determined using ANOVA and the Dunnett *post hoc* test, p < 0.05 compared with the corresponding controls.

ROS expressed as regression coefficients (in RFU) are shown in Figure 5. Silver nanoparticles exhibit production of ROS significantly different from controls from 1 mg·L^-1^ to 6 mg·L^-1^. The RFU shows significantly increased fluorescence in the case of copper nanoparticles compared to the control group at all studied concentrations, except for the highest concentrations of 800 and 400 mg·L^-1^. High concentrations of silver NPs, as well as copper NPs, strongly influence metabolic processes, resulting in rapid cell death; therefore, almost no ROS can be detected. Cells are not able to maintain basic metabolic processes and produce ROS at such high silver and copper concentrations, representing a highly toxic environment. Interestingly, Au NPs exhibit a significant reduction in ROS production compared to the control group. This phenomenon is common and well-documented in literature. It is reported that gold nanoparticles, due to their low toxicity, relative chemical inertness, and stability, can act as antioxidants or ROS neutralizers by binding free radicals when they interact with electrons in the conduction path of gold nanoparticles, thereby stopping the chain reaction of oxidative stress. In this case, gold nanoparticles can be utilized in various applications, including antioxidant protection, anti-inflammatory effects, wound healing, slowing the aging process, and treating diseases where oxidative stress plays a crucial role. However, the antioxidant effect of Au NPs depends entirely on their physicochemical parameters, such as size, shape, surface treatment, and stabilizers used, as well as the fact that different synthesis methods lead to different effects. Other factors that influence this include the concentration of nanoparticles used, exposure time, and, naturally, the type of biological model studied (Suliasih et al., 2024; Beurton et al., 2019; Aili et al., 2023; Mehanna et al., 2022).

### Mitochondrial membrane potential assay ΔΨm

**FIGURE 6:**
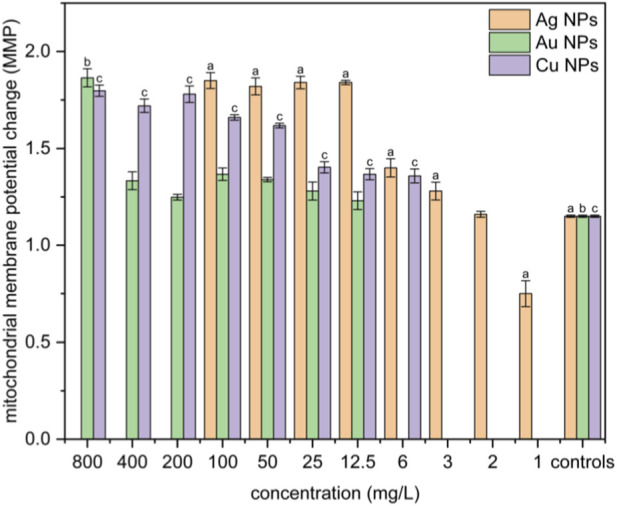
Changes in mitochondrial membrane potential in NIH 3T3 cell line elicited by Ag, Au, and Cu nanoparticles. Positive significance (a, b, c) was determined using ANOVA and the Dunnett *post hoc* test, p < 0.05 compared with the corresponding controls.

Statistical analysis revealed a significantly increased production of green monomer for concentrations higher than 3 mg·L^-1^ for Ag NPs and 6 mg·L^-1^ for Cu NPs compared to the control group, indicating more substantial mitochondrial damage and a higher probability of early-stage apoptosis at these concentrations (Figure 6). With a further increase in the Ag NPs concentration, the membrane potential remained unchanged. Copper and gold nanoparticles increased the potential of the mitochondrial membrane compared to the control group, although this effect was less pronounced than in the case of silver nanoparticles. Gold nanoparticles influenced the mitochondrial membrane potential only at the highest concentration used, which was 800 mg·L^-1^.

#### Comet assay

**FIGURE 7:**
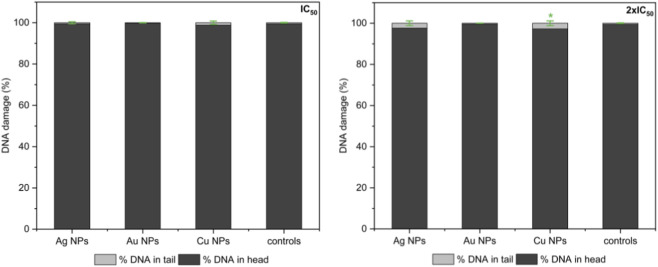
Percentage DNA in Head and in Tail determined by comet assay in NIH 3T3 cell line after application of Ag, Au, and Cu nanoparticles in IC_50_ and 2× IC_50_ concentrations. The data represent the mean and standard error from three independent measurements. Positive (*) significance was determined using the Mann–Whitney U test with Bonferroni correction for multiple comparisons significance. From left: dose equal IC_50_, dose equal 2 × IC_50_.

Comet assay was used to determine DNA fragmentation of NIH 3T3 cell line 6 h after application of metal nanoparticles at a concentration of IC_50_ and 2× IC_50_ (see Figure 7). The fluorescence intensity of the comet tail relative to the head reflects the number of DNA breaks (more comet tail length means more DNA damage). Statistical analysis confirmed that none of the compounds studied exhibited a significant effect on DNA.

#### Apoptosis and necrosis

**FIGURE 8:**
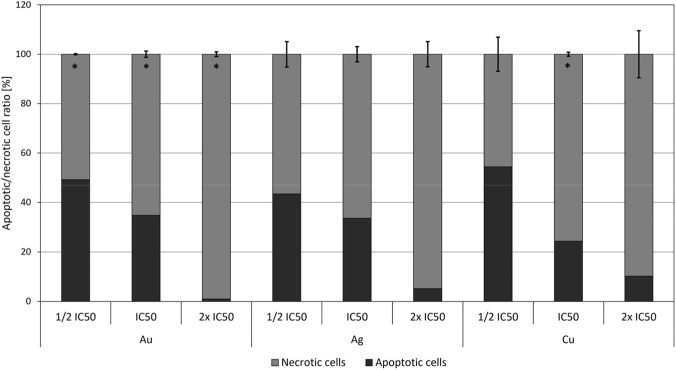
Increase in apoptotic and necrotic cells compared to control determined by fluorescence assay in NIH 3T3 cell line after application of Ag, Au, and Cu nanoparticles in ½ IC_50_, IC_50,_ and 2× NP concentrations. Positive (*) significance was determined using Student’s t-test and Bonferroni correction (*p < 0.05 compared with the corresponding controls). Statistically significant differences in the proportion of necrotic cells were observed for Au nanoparticles at ½ IC_50_ (p = 0.008), IC_50_ (p = 0.038) and 2×, IC_50_ (p = 0.024). For Cu nanoparticles, a significant increase was detected at IC_50_ (p = 0.021).

The numbers of cells undergoing apoptosis and necrosis are shown in Figure 8 as the average values for each tested nanoparticle. Three concentrations were tested for each metal: half the IC_50_ concentration, the IC_50_ concentration itself, and twice the IC_50_ concentration. The results indicate that all nanoparticles induce both apoptotic and necrotic cell death, depending on the increasing concentration of metals. The results show that low concentrations of metal nanoparticles, specifically ½ IC_50_, generally lead to a higher number of apoptotic cells compared to higher concentrations. This suggests that lower concentrations reduce toxic stress, cause less damage to cell membranes, and help maintain the cells’ ability to activate controlled apoptotic pathways. Notably, for copper nanoparticles, there is an increase in measured apoptosis at a concentration double the IC_50_. However, apoptotic cell death values are generally similar at the half and full IC_50_ concentrations, indicating a clear concentration dependence. In terms of necrosis, significant differences were observed among the metal concentrations. The same trend was seen across all nanoparticles: the number of necrotic cells increased with higher concentrations. In fact, the number of necrotic cells after exposure to double the IC_50_ concentration was several times higher compared to those exposed to half the IC_50_ or the IC_50_ concentration.

## Discussion

To compare the cytotoxicity of noble metal nanoparticles solely based on their chemical identity, we synthesized silver, gold, and copper nanoparticles under identical reaction conditions using NaBH4 as a strong reducing agent and polyacrylic acid as a stabilizer. This approach ensured the formation of small, spherical particles with a similar size distribution and surface charge, minimizing the influence of morphology, aggregation, or surface chemistry. Under these controlled conditions, the resulting dispersions differed exclusively in the chemical nature of the metallic core. Residual nitrate and chloride anions from precursor salts were removed during washing and did not affect the cytotoxicity of the nanoparticle dispersions. This strategy enabled a direct comparison of cytotoxic effects and mechanisms determined purely by metal composition. In this work, we performed a complex *in vitro* study of cytotoxic effect by determining the IC_50_ values of the tested nanoparticles of noble metals. We also described the mechanism of action of these nanoparticles, which depends on the chemical identity of nanometals only, not on their size, shape, morphology, or chemical composition of water dispersion. Metallic nanoparticles can affect various cellular components, including DNA and mitochondria, and can also trigger the production of reactive oxygen species and induce apoptosis (Wang et al., 2024; Uma et al., 2025). For these reasons, the present study also focused, among other aspects, on a broad range of assays designed to evaluate these interactions. IC_50_ values of noble metal nanoparticles were determined using the MTT assay, followed by *in vitro* assessments of potential cytotoxic mechanisms, including oxidative stress, mitochondrial membrane potential, cell death type, and genotoxicity. Each of these cytotoxic parameters indicates different toxic effects of nanoparticles, including the cytotoxic mechanism responsible for their cytotoxicity. Using a single cell line allowed us to compare the cytotoxicity induced by nanoparticles solely on the basis of their chemical composition, in a controlled manner. NIH 3T3 fibroblasts were selected as a robust and well-established *in vitro* model commonly used in nanotoxicology studies, providing reproducible results with well-characterized responses to stress and cell death. By minimizing cell-type-dependent variability, this design enabled the observed differences in cytotoxicity, oxidative stress, and cell death pathways to be attributed primarily to the intrinsic properties of the core and metal nanoparticles.

MTT assay demonstrated that Ag NPs exhibited the highest cytotoxicity (IC_50_ = 11.9 mg·L^-1^), followed by Cu NPs (IC_50_ = 51.6 mg·L^-1^) and Au NPs (IC_50_ = 228.2 mg·L^-1^). For silver, a sharp decline in NIH 3T3 cell viability occurred between 6 and 12.5 mg·L^-1^. While a concentration of 6 mg·L^-1^ has virtually no negative impact on the viability of test cells (with almost 100% viability), a concentration of approximately 12 mg·L^-1^ shows a significant decline, resulting in cell viability dropping below 50%. This study demonstrated an IC_50_ of 11.9 mg·L^-1^ for silver nanoparticles, which are approximately 10 nm in size, consistent with IC_50_ values ranging from about 2 mg·L^-1^ to 37 mg·L^-1^ for eukaryotic cells found in the literature (Yang et al., 2016; Dziedzic et al., 2016; Souza et al., 2016). Copper nanoparticles show a moderate cytotoxic effect on the tested fibroblasts in comparison with silver NPs. IC_50_ for copper NPs was determined to be 51.6 mg·L^-1^. The viability of cells treated with Cu NPs differed significantly from that of control cells at a concentration of 50 mg·L^-1^ and higher. Nevertheless, a concentration of 25 mg·L^-1^ is not toxic to treated cells, as the viability decreased to 90% only compared to control cells. The literature indicates a wide range of IC_50_ values for 10 nm copper nanoparticles, varying from 2.5 mg·L^-1^ in breast and ovarian cancer cell lines to 410 mg·L^-1^ in the HEK 293 (Human Embryonic Kidney) cell line. In our study, we observed a value that falls within this range. However, it is important to note that these results are influenced by several factors, including the method of synthesis, surface treatment, and, crucially, the biological model used in the studies (Letchumanan et al., 2021; Liang et al., 2022). Gold nanoparticles appear to be the least toxic compared to silver and copper NPs. A decrease in viability is detected only for concentrations of 100 mg·L^-1^ and higher. A high IC_50_ value (228.23 mg·L^-1^) indicates very low toxicity of gold NPs against murine fibroblasts and/or low sensitivity of these cells to gold nanoparticles. Similar values (216 ± 6 mg·L^-1^) were also published in Vijayakumar and Ganesan (2013). Other studies allude to the low toxicity and biocompatibility of gold nanoparticles (Mikhailova, 2021; Coradeghini et al., 2013; Ibrahim et al., 2023).

Oxidative stress is one of the most discussed cytotoxic mechanisms induced in cells by metal NPs (Chen et al., 2013; Ibrahim et al., 2023; Lee et al., 2014; Avalos et al., 2014; X et al., 2014; Xin et al., 2016), especially in the case of silver NPs (Bidan and Al-Ali, 2024; Zhang et al., 2022; Xin et al., 2016). The formation of reactive oxygen species and induction of oxidative stress is an attractive hypothesis to account for the mutagenic effects of metal nanoparticles in mammalian cells, as ROS are capable of causing oxidative damage to proteins and DNA. In cases of highly toxic concentrations of silver, which exceed the IC_50_ value, increased production of reactive oxygen species (ROS) was not confirmed. This may be explained by the low number of surviving cells and the fast, direct toxic effects of silver NPs. Silver nanoparticles have a significant impact, even at low concentrations ranging from 1 mg·L^-1^ to 6 mg·L^-1^, where the observed values are relatively consistent. A more pronounced reduction in ROS formation occurs at concentrations exceeding 12.5 mg·L^-1^, and this decline continues as the silver concentration increases. This confirms that high concentrations of Ag NPs induce rapid, lethal effects consisting of cell lysis and death without involving oxidative stress. These observations are consistent with the work of other researchers, who have described oxidative stress only at very low concentrations of Ag NPs (Zhang et al., 2022; Talarska et al., 2021). Our results therefore support the view that ROS contribute to early cellular stress but are not the dominant mechanisms of cell death at high Ag NP concentrations. In contrast, Cu NPs generate a strong, dose-dependent increase in reactive oxygen species (ROS) across a wide range of concentrations. Although the overall toxicity of copper to murine fibroblasts is more than five times lower than that of silver, copper still leads to significant ROS production, peaking at a concentration of 12.5 mg·L^-1^. This suggests that copper nanoparticles can elevate intracellular ROS levels in murine fibroblasts in a dose-dependent manner. However, at high concentrations, both copper and silver can disrupt the internal environment of the cells, making it difficult to demonstrate ROS production. These findings are consistent with existing literature, which indicates that the toxicity of Cu NPs is largely mediated by ROS (Naz et al., 2020; Baeg et al., 2018; Kumari et al., 2017). Gold nanoparticles exhibited distinctly different behavior by reducing ROS levels below control values while maintaining similar levels across all measured concentrations (12.5–800 mg·L^-1^). This clearly indicates that concentration does not influence ROS production in the case of gold. This observation is consistent with several studies reporting minimal or even decreased ROS generation by Au NPs (Liang et al., 2022; Lee et al., 2014). Thus, our results support the established view that Au NPs are chemically inert and induce negligible oxidative stress.

Since oxidative stress can affect membrane potential, cell aging, and cell death, we examine the changes in mitochondrial membrane potential of cells treated with metal nanoparticles. These changes in mitochondrial membrane potential closely mirrored the trends in reactive oxygen species results. Silver nanoparticles had a slight effect on the membrane potential at concentrations of 2 mg·L^-1^, but significant changes were observed at 3 mg·L^-1^ and higher. Mehmood et al. (2025) reported that mitochondrial damage and dysfunction in fibroblasts occur at Ag NP concentrations of approximately 20 μg·mL^-1^ and higher, which is in accordance with our findings. This is also consistent with MTT assay results, which showed a marked decrease in cell viability at IC_50_ values and above. For concentrations above 12 mg·L^-1^, the membrane potential remained relatively constant, as cell viability decreased below 10%. There is a direct correlation between the increased production of ROS and a significant change in the membrane potential at concentrations corresponding to the IC_50_. Copper NPs induced a clear concentration-dependent decline in membrane potential across the full concentration range, consistent with ROS-mediated mitochondrial damage. This observation is in agreement with the findings of Wang et al. (2025), who reported that elevated levels of copper ions lead to mitochondrial dysfunction. Copper catalyzes the formation of ⋅OH from H_2_O_2_ via the Fenton reaction, resulting in direct damage to mitochondrial membrane lipids, proteins, DNA and ultimately leading to mitochondrial impairment. Gold nanoparticles have a minimal effect on mitochondrial membrane potential, significantly increasing it only at the highest concentration of 800 mg·L^-1^. This aligns with findings from Aueviriyavit et al. (2014) and is consistent with data from viability and ROS tests.

The comet assay determines DNA fragmentation in the NIH 3T3 cell line 6 h after the application of nanoparticles at concentrations of IC_50_ and 2× IC_50_ and monitors the percentage of DNA in the tail compared to the control. NIH 3T3 cells treated with Ag nanoparticles exhibited a dose-dependent increase in DNA breakage. For silver in a concentration equal to the IC_50_, there was not observed significant difference versus the controls. For twice the higher concentration (2× IC_50_), the amount of DNA in the tail increased 4.3–fold relative to controls. However, even in this case, there is no statistically significant difference, and the genotoxic effect of silver nanoparticles at the 2× IC_50_ dose can be excluded. Similar values were obtained by Kim et al. (2013) in a mammalian cell system cells and Nam et al. (2013), who did not confirm genotoxicity for the citrate–capped Ag NPs (size 10 nm). Regarding the influence of copper nanoparticles on DNA, we observed a concentration-dependent effect, where higher concentrations resulted in greater DNA damage. Midander et al. (2009) investigated the effect of micro and nanoparticles of copper on DNA and mentioned a significant effect of 100 nm nanoparticles (concentration 80 mg·L^-1^) on the DNA. The increase was 4.3 times that of controls, which also corresponds to our observation. Midander points out that the cytotoxicity induced by nanosized copper can be caused by nanoparticles, and only a small extent of the released ions is responsible. As mentioned above, gold NPs do not significantly affect cell viability, do not participate in ROS production or changes in membrane potential, and generally appear to be the least toxic of the three compared metal NPs. However, if we turn our attention to the influence of the Au NPs on DNA, the situation is different. Lower concentration (IC_50_) with only 0.29% DNA in the tail has virtually no effect on DNA, and also a double dose of 2× IC_50_ with 0.41% DNA in the tail causes negligible damage to DNA. Even here, we cannot discuss genotoxicity, which is confirmed by other studies (Nam et al., 2013; Chueh et al., 2014). The lack of statistical significance (p > 0.05) in the increase in DNA in the tail section for AgNPs (2.38% ± 1.12% at 2× IC50, 4.3-fold compared to the control) and AuNPs (2.84% ± 0.18%, 5.2-fold) may be attributed to high variability between cells (SD 0.18%–1.12%), reflecting heterogeneous nanoparticle uptake and DNA repair in primary cells. Another factor may be the threefold biological replicate, which is less sensitive to subtle changes of less than 3%. Biological insignificance may be another important factor, as absolute DNA levels in the tail remained <3%, well below genotoxicity thresholds. In contrast, CuNPs achieved significance (p < 0.05) due to larger effect sizes that exceeded variability.

The results of the Comet assay are supplemented by interesting observations of cell death monitoring. For all three types of metallic nanoparticles, exposure at half the IC_50_ concentration resulted in a higher proportion of apoptotic cells compared to higher concentrations. In fact, increased concentrations led to a significant shift towards necrosis as the predominant mode of cell death. Xi et al. (2023) described the mitochondrial permeability transition–driven necrosis. This represents a form of regulated cell death that is activated, among other factors, predominantly by oxidative stress. This leads to a sudden loss of mitochondrial membrane potential and thus providing an additional explanation for why an increasing loss of membrane potential is associated with a higher proportion of necrotic cells. While all three nanoparticles displayed a trend of increasing necrotic cell proportions with higher concentrations, specific differences were evident when comparing particular concentrations. Copper nanoparticles exhibited the highest proportion of apoptotic cells at a concentration of ½ IC_50_. Conversely, at the IC_50_ concentration, the lowest proportion of apoptotic cells was observed, with a higher proportion of necrotic cells at this level. Copper nanoparticles increase the proportion of apoptotic cells, even at higher concentrations, and this finding correlates with the marked increase in ROS levels associated with copper. This indicates that the cytotoxic effects of copper at elevated concentrations are mainly driven by ROS-mediated apoptotic signaling. At concentrations twice that of IC_50_, necrosis predominated almost entirely, particularly with gold nanoparticles, which had the highest proportion of necrotic cells at this dosage. Across the tested concentrations, gold nanoparticles consistently induced a significantly lower proportion of apoptotic cells accompanied by an increased necrotic response compared to the control, with this effect observed at ½ IC50, IC50 and 2× IC_50_. In contrast, a similar shift from apoptosis toward necrosis was detected for copper nanoparticles only at the IC50 concentration. These findings suggest that sub-cytotoxic doses promote regulated (programmed) cell death, while exposure to higher concentrations of metal nanoparticles leads to extensive loss of membrane integrity and uncontrolled necrosis. This highlights the critical role that concentration plays in determining the nature of cytotoxicity induced by nanoparticles.

Silver, gold, and copper nanoparticles were synthesized under identical conditions, meaning that the observed differences in cytotoxicity can be attributed primarily to the intrinsic properties of the metal cores. The MTT assay showed a clear cytotoxicity trend Ag > Cu > Au. Silver nanoparticles induced early oxidative and mitochondrial stress already at sub-IC_50_ concentrations, while higher doses led to rapid loss of membrane integrity and predominantly necrotic cell death, accompanied by reduced measurable ROS levels due to extensive cellular damage. Copper nanoparticles exhibited moderate toxicity, characterized by a dose-dependent increase in ROS and a marked decrease in mitochondrial membrane potential, which promotes apoptosis at lower doses and necrosis at higher concentrations. Gold nanoparticles were the least toxic and caused minimal ROS generation, only minor alterations in mitochondrial membrane potential, and no detectable genotoxicity. Even at elevated concentrations, their effects were consistent with passive physical or metabolic stress rather than a specific biochemical mechanism of toxicity.

## Conclusion

Our study evaluates the cytotoxic effects of silver, gold, and copper nanoparticles on the NIH 3T3 mouse fibroblast cell line. We aimed to compare these effects among the nanoparticles, all of which were prepared under identical conditions with similar shapes, sizes, and surface charges. This ensures that any observed differences in their biological effects are due solely to their chemical composition. This study employed a controlled comparison of the cytotoxic effects of silver, copper, and gold nanoparticles on a single fibroblast cell line, reducing variability caused by different cell responses. While the results clearly show toxicity patterns dependent on composition, further research with multiple cell types is needed to evaluate broader biological significance. The cytotoxic effects of metal nanoparticles are dose-dependent. Higher doses consistently increase cytotoxicity across all nanoparticles. Copper nanoparticles are more toxic at higher doses, while silver nanoparticles can kill cells at lower concentrations than gold or copper nanoparticles. Necrosis is predominant at high doses, while apoptosis is more prominent at lower concentrations. This trend is consistent across all the metals. All types of metal nanoparticles induce oxidative stress, leading to cell death through multiple mechanisms. DNA damage assessments indicate that cells exposed to all three types of nanoparticles exhibit increased DNA fragmentation, which is indicative of both apoptosis and necrosis. The findings from the Comet assay and cell death monitoring provide complementary evidence in this context. The higher surface area-to-volume ratio of nanoparticles around 10 nm increases their chemical reactivity and, consequently, their cytotoxicity as the size decreases. A promising outcome of our study is that despite the increased cytotoxicity of Ag and Cu nanoparticles, no mutagenic or genotoxic effects were observed in the tested fibroblast cell line. Future research should investigate long-term exposure to assess bioaccumulation and cellular adaptations. A comparative analysis of cytotoxic responses in various mammalian cell lines, including human cells, will help identify species-specific sensitivities and biocompatibility. Studying the effects of surface modifications on nanoparticle toxicity could help develop safer materials for biomedical use. Expanding research to *in vivo* models would provide a deeper understanding of nanoparticle toxicity and its systemic effects, thereby guiding the safe application of nanoparticles in medicine.

## Data Availability

The original contributions presented in the study are included in the article/supplementary material, further inquiries can be directed to the corresponding authors.
